# A Case Study on Attribute Recognition of Heated Metal Mark Image Using Deep Convolutional Neural Networks

**DOI:** 10.3390/s18061871

**Published:** 2018-06-07

**Authors:** Keming Mao, Duo Lu, Dazhi E, Zhenhua Tan

**Affiliations:** 1 College of Software, Northeastern University, Shenyang 110004, China; lu.d.0@foxmail.com (D.L.); tanzh@swc.neu.edu.cn (Z.T.); 2 Shenyang Fire Research Institute, Ministry of Public Security, Shenyang 110034, China; edazhi@syfri.cn

**Keywords:** attribute recognition, heated metal mark, convolutional neural networks

## Abstract

Heated metal mark is an important trace to identify the cause of fire. However, traditional methods mainly focus on the knowledge of physics and chemistry for qualitative analysis and make it still a challenging problem. This paper presents a case study on attribute recognition of the heated metal mark image using computer vision and machine learning technologies. The proposed work is composed of three parts. Material is first generated. According to national standards, actual needs and feasibility, seven attributes are selected for research. Data generation and organization are conducted, and a small size benchmark dataset is constructed. A recognition model is then implemented. Feature representation and classifier construction methods are introduced based on deep convolutional neural networks. Finally, the experimental evaluation is carried out. Multi-aspect testings are performed with various model structures, data augments, training modes, optimization methods and batch sizes. The influence of parameters, recognitio efficiency and execution time are also analyzed. The results show that with a fine-tuned model, the recognition rate of attributes metal type, heating mode, heating temperature, heating duration, cooling mode, placing duration and relative humidity are 0.925, 0.908, 0.835, 0.917, 0.928, 0.805 and 0.92, respectively. The proposed method recognizes the attribute of heated metal mark with preferable effect, and it can be used in practical application.

## 1. Introduction

With the rapid development of construction industry and material technology, metal components are widely used in modern architecture and domestic appliance. Generally, metal components are nonflammable and can be retained after the fire. When being heated, complex physical and chemical changes happened on the metal component. Consequently, various marks are left on the surface of the metal component. The heated metal mark is influenced by attributes of heating temperature, heating duration, heating mode, cooling mode, etc. The oxidation reactions on surface of metal are different with various attribute conditions. These attributes of heated metal marks are useful clues to locate the fire point, and then the source and situation of fire could be further analyzed. The scene of the fire is very complicated and cannot reappear. Therefore, it is a better way to recognize or classify attributes of heated metal through observing its marks image.

Table 1 gives the inspection methods for trace and physical evidences from fire scene (a National standard of People’s Republic of China) [1]. It includes relationship between color of heated metal mark and heating temperature. It should be noted that the color range of metal is determined by human expert. According to the standard, Ying Wu et al. utilized metal oxidation theory to analyze the relation between color of metal surface and its heating temperature. When heating temperature approaches or exceeds melting point, the metallographic organization significant changed [2,3]. Zejing Xu and Yupu Song proposed a method to record attributes value of object surface by means of macro-inspection and micro-analytical. Then fuzzy mathematics was adopted to establish temperature of building component [4]. Dadong Li and Tengyi Yu analyzed the changes in the surface of Zn-Fe alloy with different temperature and heating duration. By using stereo microscope and electron microscope, they found that chemical composition and organization structure were changed and these leaded to the color change on the surface of metal [5].

It can be seen that traditional methods for this problem are mainly based on the knowledge of physics and chemistry for qualitative analysis. However, it is usually unpractical to implement and with less automation. This paper takes another point of view, completely relies on computer vision and machine leaning technologies. The attributes of heated metal are modeled and analyzed by data-driven mode and intelligent recognition method is devised.

Image recognition is a classical problem in computer vision and machine learning fields. With annotated training dataset, supervised learning or unsupervised learning method can be adopted. It has two main steps. First, features are extracted from training images. Second, classifier models are trained with feature vectors and corresponding attribute labels.

Image feature representation is a key research field and many works have been reported. Before the year 2012, mainstream methods for image feature extraction and representation are based on hand-craft features by experienced scientist and engineer. Image local feature extraction and representation algorithms are designed to deal with content translation, scale variant, rotation, illumination and distortion, as much as possible. Local image descriptors are then transformed into feature vectors, and global image feature representation is aggregated with all local feature vectors. Some representative researches are introduced in the following statements. David Lowe proposed SIFT (scale invariant feature transform descriptor) [6,7]. It was computed from the pixel intensity around a specific interest point in image domain. A SIFT descriptor was encoded with 4×4×8=128 dimensions for each interest point. Dalal and Triggs developed an image local descriptor HOG (histograms of oriented gradients) which was computed from a group of gradient orientation histograms within subregions [8]. The dimension of HOG descriptor was determined by the number of cells per block, the number of pixels per cell and the number of channels per cell histogram. The SURF (speeded-up robust features) descriptor proposed by Bay et al. was closely related to the SIFT [9]. The main difference was that SURF was computed based on Haar wavelets and the interest point was determined based on approximations of scale-space extrema of the determinant of the Hessian matrix. SURF had better computational efficiency. Gaborfilter was a linear filter that commonly used in image texture analysis [10,11]. A classical 2D Gaborfilter in spatial domain can be seen as a sinusoidal plane wave modulated by Gaussian kernel, and whether there were any specific frequency content with the specific directions in a localized region of an image can be estimated. LBP (Local binary patterns) was another powerful feature descriptor for image classification [12]. It was computed based on comparison between a pixel with each of its 8 neighbor pixels. It defined an 8-digit binary number in clockwise or counter-clockwise orientation. The frequency of each binary number was computed and the final feature vector was represented by accumulating all cells in a region. Moreover, various improvement versions of these local feature descriptors were proposed constantly.

Image global descriptor is then represented based on these local feature descriptors. BoVW (bag of visual words) model was one of most widely adopted methods [13]. First, visual words were gained by clustering all local feature vectors and visual vocabulary was comprised of all visual words. Then each local patches of an image can be mapped to a visual word and the whole image was represented by the histogram of the visual word frequency. One disadvantage of original BOVW model was that it lacked spatial relationship of image content. Kristen Grauman and Trevor Darrell proposed SPM (spatial pyramid matching method) [14]. SPM treated an image as multi-resolutions, and it generated histograms by binning data points into discrete regions of different size. Thus, features that did not match at high resolutions can also be matched at low resolutions. VLAD (Vector of Locally Aggregated Descriptors) [15] and FV (Fisher Vector) [16] methods were presented that based on encoding the first and second order statistics of feature vector. They not only increased classification performance, but also decreased the size of visual vocabulary and lowered the computational effort.

With the global image feature representation, metrics between high dimension feature vectors are used to measure difference between images object. SVM (Support vector machine) was the most widely used classifier training method [17]. It treated features as points in high dimensional space and mappings was conducted that the examples of the separate categories were divided by hyper-planes which was forced as wide as possible.

Many public available image benchmark datasets were provided to speed up the technology development with large size labeled training samples. ImageNet and COCO were the two most famous sets. ImageNet was first opened by Jia Deng et al. in the year 2009 [18]. It contained at least 14 million images and covered over 20,000 categories. Microsoft COCO dataset was opened in 2014 and with a total of 2.5 million labeled instances in 328,000 images [19]. These datasets not only provided large size labeled images, but also provided platforms for comparison of different algorithms based on the unified standards.

Recently, deep learning has scored great success in machine learning field especially for image classification [20]. It is also called deep structured learning or hierarchical learning and essentially it is a special form of neural network. It uses a cascade of multiple layers of nonlinear processing units for feature transformation and extraction. The main advantages of deep learning are: (1) Feature extraction in deep level. It generates compositional models where the object is expressed as a layered composition of primitives; and (2) efficient parameter adjustment. The parameters in deep model for feature extraction are tuned based on training data and loss function completely automatic. Yan LeCun designed a small scale convolutional neural networks, LeNet, with the purpose of recognizing handwritten mail ZIP code [21,22]. A medium scale deep convolutional neural networks, AlexNet, proposed by Krizhevsky and Hinton won the ImageNet competition by a significant margin over traditional methods [23]. In the next few years, several more powerful models were proposed. ZFNet, VGGNet, GoogleNet and ResNet won the ImageNet image classification competition successively [24,25,26,27]. ResNet achieved an excellent top-5 error performance with 3.57% and outperformed humanity for the first time.

According to our knowledge, there is no researches focus on our problem. Some most relevant works are reviewed. A rail surface defects type detection method was proposed [28]. It constructed a deep network with three convolutional layers, three max-pooling layers and two fully connected layers. Twenty-two thousand four-hundred eight object images were manually labeled. Using the larger network and 90% percent data for training, 92.47% multi-class accuracy was obtained. A bearing fault diagnosis algorithm was introduced based on ensemble deep networks and an improved Dempster–Shafer theory [29]. Models used in this work was a smaller one with 3 convolutional layers and 1 fully connected layer. This fusion model combined multiple uncertain evidences and computed the result through merging consensus information and excluding conflicting information. Ten thousand image samples were used for training and 2500 image samples were used for testing. With fusion and ensemble, it gained 98.72% performance for 10-type fault type classification. A deep learning-based method was proposed for characterization of defected areas in steel elements with utilization of a magnetic multi-sensor matrix transducer and integration of data [30]. In this method, three united architectures for multi-label classification were used for evaluation of defect occurrence, rotation and depth. Basiclly, this model contains three convolutional layers, three max-pooling layers and one fully connected layer. Thirty-five thousand simulated data samples were generated. Data used for training and testing was set with a ratio of 85:15. A surface defects classification method was proposed for hot-rolled steel sheet [31]. The network contained seven layers, and eight surface defects were defined. There were 14,400 samples for the whole dataset and 1800 samples for each type. Ninety-four percent accuracy was obtained with 5/9 data for training. A damage detection method of civil infrastructure was designed [32]. The model contained three convolutional layers, three pooling layers and one fully connected layer. The images were divided into small patches, and were manually annotated as crack or intack. The dataset contained 40,000 samples, and 90% used for training. 98.22% accuracy performance was obtained with sliding windows. A Faster*r*-cnn-based method was used for structural surface damage detection [33]. 5 types of surface damage were defined as concrete cracks, steel corrosion (medium and high levels), bolt corrosion, and steel delamination. ZFNet was used as the backbone network. Two-thousand three-hundred sixty-six image samples were collected as the dataset. This model achieved a 87.8% accuracy with 2.3:1 proportion of training and testing samples. A multilevel deep learning model was proposed for surface defect and crack detection inside steel box girder [34]. This model included three bypass to concatenate the final feature representation. Three types, including crack sub-image, handwriting sub-image, background sub-image were defined. Raw images were obtained by common digital camera. After division, 67,200 sub image samples were generated. With 80% dataset for training, 95% mean accuracy precision was obtained. Moreover, the effects of super-resolution inputs were also investigated. These related works made similar studies to the proposed one. However, these methods usually adopted relatively simple models and the state-of-art deep learning models were not concerned. The training and optimization procedure were not demonstrated clearly. Based on these points, we carry out our research.

**Figure 1:**
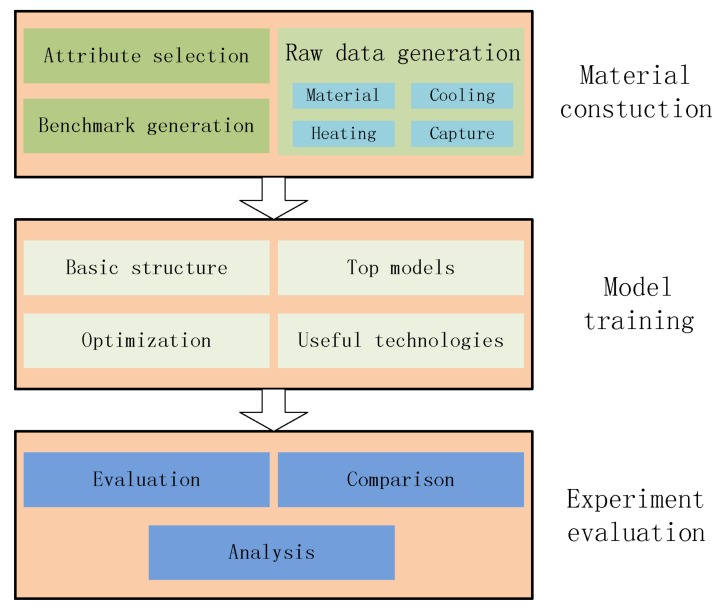
The framework of this work.

This paper presents a case study on heated metal attribute recognition by deep convolutional neural networks model. There are three important stages: (1) Material construction stage. Attributes of heated metal are first defined as needed. Then the procedure of raw image data generation is designed, including material type, heating mode, cooling method and capture device, etc. Benchmark dataset is finally organized; (2) Model training stage. Deep convolutional neural networks models used in this work are introduced, including basic structure, top models structure and useful technologies; (3) Experimental evaluation stage. Experiments and analysis are carried in many aspects, including performance on different models, parameters setting, data augment, model convergence, recognition efficiency and execution time. Figure 1 gives the whole framework of this study.

The main contributions of this paper are threefold:

- Deep convolutional neural networks models are adopted to recognize attribute of heated metal based on its marks image;
- The material benchmark dataset is completely new designed and generated;
- Extensive experimental evaluations and analyses are carried out.

The rest of this paper is organized as follows. Section 2 presents the materials generation. Section 3 describes the methodology. Experimental evaluation and analysis are given in Section 4. Section 5 concludes this paper.

## 2. Materials Generation

Since there are no benchmarks in related fields, dataset for training and testing is constructed in this work. This section includes attributes definition, raw image generation and benchmark dataset construction.

### 2.1. Attribute Definition

According to the conditions defined in National standard of People’s Republic of China GB/T42327905.3-2011 (inspection methods for trace and physical evidences from fire scene—Part 3: Ferrous metal work) [1], the heating temperature of metal is the the most important factor. In addition, other important factors are also covered for practical demands. Therefore, metal types, heating mode, heating temperature, heating duration, cooling mode, cooling humidity and placing duration are used as basic attributes which we want to recognize from heated metal mark image. The attributes are configured as follow:

- Metal type. This attribute indicates the type of heated metal in the fire scene;
- Heating mode. This attribute indicates heating source and form;
- Heating temperature. This attribute indicates the temperature degree of the metal that being heated;
- Heating duration. This attribute indicates the duration time of the metal that being heated;
- Cooling mode. This attribute indicates the method of the heated metal that being cooled;
- Cooling humidity. This attribute indicates the humidity degree when the heated metal that being cooled;
- Placing duration. This attribute indicates the duration time of the heated metal that being cooled.

For each attribute, its value ranges considered in this study are detailed described in Table 2. For simplicity, attribute *i* is abbreviated as $a_{i}$ in the subsequent sections.

### 2.2. Raw Image Generation

**Figure 2:**
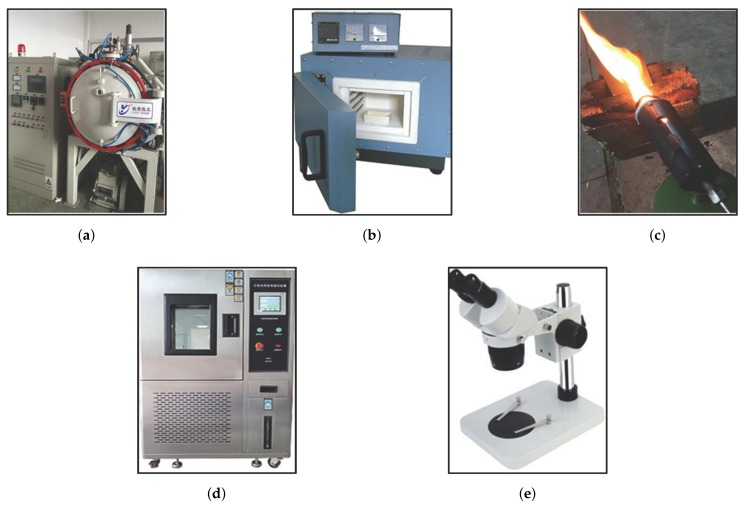
Devices used in this study for generating image dataset. (**a**) demonstrates vacuum resistance furnace; Muffle furnace and gasoline burner are shown in (**b**,**c**) respectively; (**d**) is a test chamber with constant temperature and humidity.; Heated metal mark images are captured via microscope in (**e**).

Two widely used metal materials, galvanized steel and cold rolled steel, are selected as research objects. The metal plate is first cut to equal size (length = 1.0 cm, width = 1.0 cm, thickness = 1.0 mm). This guarantees the consistency of the experimental conditions. Three devices, a vacuum resistance furnace, muffle furnace and gasoline burner, are used to heat metals for simulating three different heating scenes. Figure 2a–c demonstrate the three devices respectively. After heating to a specific temperature ($a_{3}$) and duration time ($a_{4}$), the metals are placed in a test chamber, as shown in Figure 2d. The test chamber provides constant temperature and humidity, so attributes of cooling mode ($a_{5}$), placing duration ($a_{6}$) and relatively humidity ($a_{7}$) can be employed. To exhibit better appearance feature of sample images, we do not use traditional camera, instead a special purpose microscope is used to capture the heated metal mark image, as shown in Figure 2e.

### 2.3. Benchmark Dataset Construction

**Figure 3:**
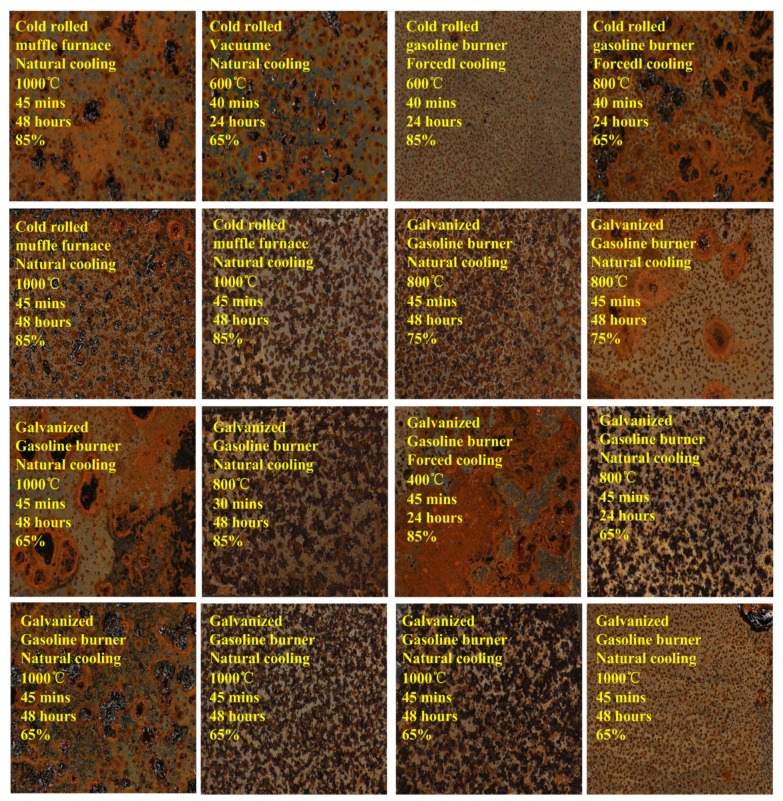
Demonstration of generated heated metal mark image samples. 7 attributes are labeled at the top-left position of each image.

According to the conditions and processes set up above, independent productions are conducted. The image sample is captured with a resolution of 2152×1616 pixels. Each heated metal mark image sample is labeled with 7 attribute values as illustrated in Table 2. Image samples are demonstrated in Figure 3, and totally there are 900 image samples. Based on the generated image dataset with attribute label values, this work makes a case study to analysis and construct relations between heated metal mark image and its attributes based on computer vision and deep convolutional neural networks model.

## 3. Methodology

In this study, we want to design a model that can predict metal attribute based on its mark image. The basic formation can be written as Equation (1). *x* denotes a heated metal mark image. *y* is the attribute value estimated by a classifier model f(). In the following, basic structures of convolutional neural networks, top CNNs models, useful techniques and pesudocode are explained.

(1) y=f(x)

### 3.1. Basic Structures in CNNs

CNNs (Convolutional neural networks) are a special form of neural network, and proved to be the most powerful model for computer vision, especially for image classification and object detection [20]. Classical CNNs are composed of three principle layers, the convolutional layer, pooling layer and fully connected layer, respectively.

#### 3.1.1. Convolutional Layer

The convolutional layer is the core building block of CNNs. It contains a set of trainable filters. Typically, the filter slides over the image spatially, and the final feature map is computed by convolution operation (dot product operation) across the whole image. Equation (2) gives the basic convolution operation. The convolution is an elementwise multiplication and sum of a filter in local image region. $con_{feature}\left[i,j\right]$, c[i,j] and I[i,j] represent convolution result, convolution filter kernel and image at indices *i* and *j*. The height and width of filter kernel is denoted by *l*. After convolution, there is always an activation operation for model simulation and optimization. Equation (3) gives the ReLU (Rectified linear units) function, one of the most popular activation function for CNNs [35]. *z* means the result of convolution. r(z) denotes the activation value and all the activation results constitute the feature map.

(2) $$conv_{feature}\left[x,y\right]=\sum_{u_{1}=-l}^{u_{1}=l}\sum_{u_{2}=-l}^{u_{2}=l}c\left[u_{1},u_{2}\right]*I\left[x-u_{1},y-u_{2}\right]$$

(3) r(z)=max(0,z)

**Figure 4:**
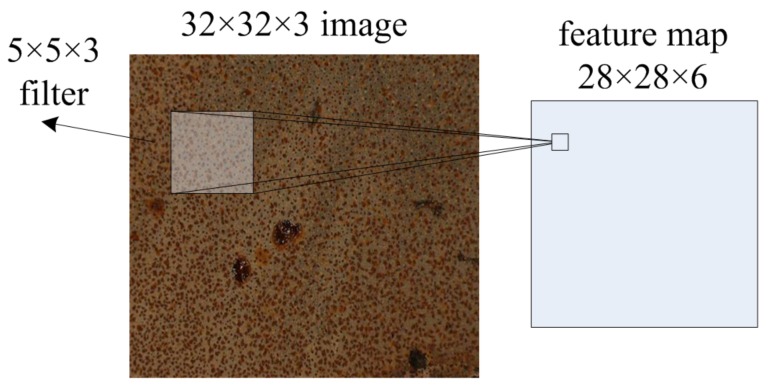
Demonstration of convolution operation in an image.

Figure 4 demonstrates the basic convolution operation of an image. Let the input image be set with 32×32 pixels and with RGB channels. It can be represented as a formation of 32×32×3 matrix. If there are 6 filters with size 5×5×3, then 6 separate activation feature maps with a stack of size 28×28×6 (with ReLU activation function, no padding, 1 pixel stride) are computed.

Ideally, one filter corresponds to a specific feature. The advantage of convolutional layer is that the local structure of an image can be captured and the parameter of a filter can be shared.

#### 3.1.2. Pooling Layer

**Figure 5:**
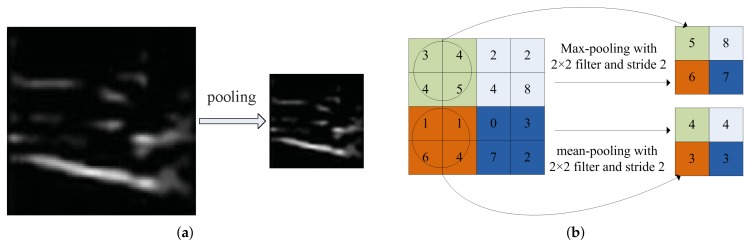
Demonstration of pooling operation.

The aim of pooling layer is to reduce the dimension of a feature map while the important feature can also be retained. It makes the feature representation smaller and more compact. The result of pooling layer is shown in Figure 5a. The basic operation of pooling is to slide a window with specified size and stride on a feature map, and the corresponding value is computed by max or mean operation inside the window, as is shown in Figure 5b. Pooling layer decreases the scale of feature map and the subsequent computation is also reduced. Moreover, pooling also reduces the number of parameters, and makes the model invariant to transformation, distortion, translation and scale change.

#### 3.1.3. Fully Connected Layer

A fully connected layer can be seen as a traditional multi-layer perceptron. Fully connected means all nodes in the previous layer are connected with all nodes of the next layer. It has two basic effects: (1) fully connected layer is another way of learning non-linear combination between features of different depth; (2) it can be used as output layer that the last feature map will be transformed into classification result with full connection. In this way softmax activation function is usually adopted.

**Figure 6:**
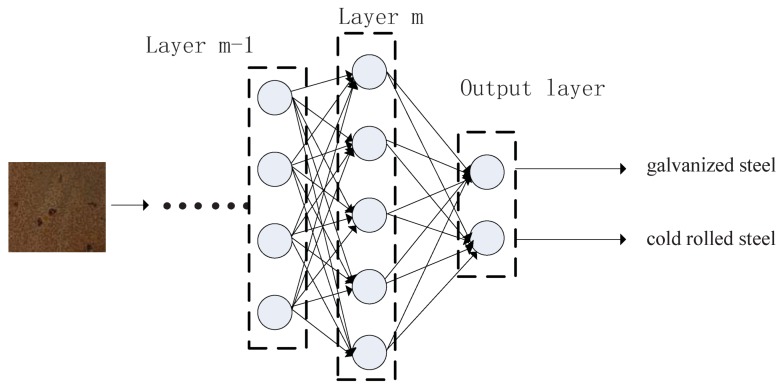
Demonstration of fully connected layers in CNN.

Figure 6 demonstrates the fully connected layer. As shown in the figure, an image is used as an input of CNNs model. Layer m−1 and layer *m* are two continuous hidden layers, which are fully connected. Meanwhile, there are two nodes in the output layer, which represents 2 attribute values of metal type that the model predicted.

#### 3.1.4. Loss Function and Model Training

Let $\left\{\left(x_{1},y_{1}\right),\left(x_{2},y_{2}\right),\ldots ,\left(x_{m},y_{m}\right)\right\}$ denote the training image data set. $x_{i}$ denotes the heated metal marks image with size w×h×c, where *w*, *h* and *c* denote the width, height and channel of input image. $y_{i}\in \left\{0,1,\ldots ,n\right\}$ is attribute label value.

For an input heated metal marks image sample data $x_{i}$, we want to compute the probability value $p\left(y=j|x_{i}\right)\left(j\in 0,1,\ldots ,n\right)$. The output, a n-dimensional vector is estimated to represent the probability of each attribute type that $x_{i}$ belongs to. The hypothesis function can be expressed as Equation (4).

(4) $$h_{\theta }\left(x_{i}\right)=\left[\begin{array}{c} p(y_{i}=0|x_{i};\theta )\\ p(y_{i}=1|x_{i};\theta )\\ \vdots \\ p(y_{i}=n|x_{i};\theta ) \end{array}\right]=\frac{1}{\sum_{j=0}^{n}\epsilon^{\theta_{j}^{T}x_{i}}}\left[\begin{array}{c} \epsilon^{\theta_{0}^{T}x_{i}}\\ \epsilon^{\theta_{1}^{T}x_{i}}\\ \vdots \\ \epsilon^{\theta_{n}^{T}x_{i}} \end{array}\right]$$

where $\theta =\{\theta_{0},\theta_{1},\ldots ,\theta_{n}\}$ is the model parameters. $\theta_{i}$ is the parameter that belongs to *i*th predicted attribute. This equation normalizes the result and makes the sum to 1. For model training, the loss function can be given as follows:

(5) $$J\left(\theta \right)=-\frac{1}{m}\left[\sum_{i=1}^{m}\sum_{j=0}^{3}1\left\{y_{i}=j\right\}log\frac{\epsilon^{\theta_{j}^{T}x_{i}}}{\sum_{l=0}^{3}\epsilon^{\theta_{l}^{T}x_{i}}}\right]+\frac{\lambda }{2}\left||\theta |\right|$$

As shown in Equation (5), formula 1{.} represents an indicative function, and the second part is a commonly used term for model regularization. Loss function usually indicates the difference between predicted attribute label and true attribute label values. The goal is to make the loss function minimal. SGD (stochastic gradient descent) method is used for optimization and the corresponding derivative functions are given as Equations (6) and (7).

(6) $$\nabla_{\theta_{j}}J\left(\theta \right)=-\frac{1}{m}\sum_{i=1}^{m}\left[x_{i}\left(1\left\{y_{i}=j\right\}-p\left(y_{i}=j|x_{i};\theta \right)\right)\right]+\lambda \theta_{j}$$

(7) $$p\left(y_{i}=j|x_{i};\theta \right)=\frac{\epsilon^{\theta_{j}^{T}x_{i}}}{\sum_{l=0}^{3}\epsilon^{\theta_{l}^{T}x_{i}}}$$

### 3.2. Top CNNs Models

In this subsection, some state-of-art CNNs models used in our study are introduced.

#### 3.2.1. VGGNet

VGGNet was introduced by Karen Simonyan and Andrew Zisserman in Visual Geomrtry Group, University of Oxford [25]. This work first explored the feasibility of increasing the depth of CNNs model with very small convolution filters (3×3 receptive field and up to 19 weight layers) for large scale image classification task. The performance on the ImageNet challenge demonstrated the effectiveness of VGGNet model.

#### 3.2.2. ResNet

Kaiming He et al. proposed ResNet in Microsoft Research [27]. This model focused on training extreme deeper networks. For solving the problem of gradient vanishing, a residual learning framework was devised. The weight layers were computed by addition with traditional stacked layer and a shortcut connection perform identity mapping. The deep residual model stacked basic building block of residual learning and made the model extremely deep (up to 152 layers). The experimental results on the ImageNet dataset demonstrated that the seemingly simple technique make the extremely deep model easier to optimize. It gained 1st place on the ILSVRC 2015 classification task while it still had lower complexity.

#### 3.2.3. Inception

This model was first introduced by Christian Szegedy et al. in Google Inc. [26]. It increased both the depth and width of the network while keeping the model computational budget constant based on Hebbian principle and multi-scale process. They devised the Inception block as a new organization. The filters were multiple scales(1×1,3×3 and 5×5). 1×1 convolutions were used as bottleneck to reduce high dimension. A 22 layers deep network was finally constructed by stacking these Inception modules. This model was referred as Inception-v1.

Sergey Ioffe and Christian Szegedy referred to the problem of internal covariate shift in deep CNNs model training [36]. They addressed it by normalizing the input of each layer. This enabled training with much higher learning rates and cared less about parameter initialization. This model was called Inception-v2.

Christian Szegedy and Vincent Vanhoucke explored to scale up networks in order to utilize the added computation as efficient as possible by suitably factorized convolutions and aggressive regularization [37]. A highest quality version of Inception-v3 was designed with better performance.

Christian Szegedy and Sergey Ioffe et al. combined the Inception architecture and Residual connections [38]. The empirical results clearly showed that the new model accelerate the network training significantly and outperformed the traditional Inception model in performance. This new model was referred as Inception-v4.

#### 3.2.4. Mobilenet

Aiming at deploy deep learning model into computationally limited platform such as mobile and embedded systems, Andrew G. Howard and Menglong Zhu et al. proposed MobileNets [39]. Depth-wise separable convolutions were introduced to build light weight deep neural networks. Trade off between model latency and accuracy were considered. While, according to the constraints of the problems, model size could be adjusted automatically.

### 3.3. Useful Technique

Here we introduce some useful techniques used for the training and optimization of deep learning models.

#### 3.3.1. Dropout

Deep convolutional neural networks usually contains a huge number of parameters, and it is prone to model overfitting. Dropout is a technique that a node is dropped out with probability of 1−p or kept with probability *p*. Only the retained nodes are trained. All dropped out networks are averaged in testing stage. This method essentially cuts node interactions, and makes the model learn better feature representation that can generalize new data. Dropout does not just decrease model overfitting, but also improve training efficiency [40].

#### 3.3.2. Data Augment

Most applications are faced with the problem of lacking sufficient training data, which is a key point for training large scale deep learning models. Data augment is a widely used method to generate new data with perturb existing one [41,42]. This method can provide more training data, reduce overfitting and improve generalization to a certain extent.

#### 3.3.3. Pre-Trained Model

Another way to manage insufficient training data is to use an existing model for initialization. Loading these parameters into the network and start to train a new one [43]. The pre-trained models are often trained with other large dataset of related domains or in-domains.

### 3.4. Pseudocode

In this subsection, Algorithm 1 is given to demonstrate the pseudocode of the proposed method. Given $Train_{set}$, $Test_{set}$ and initialized model parameter θ. A batch of sample images is random selected from $Train_{set}$. After augment, the model is trained once with *S* and parameter θ is updated. Loss *L* is computed based on $Test_{set}$. If *L* is less than ϵ or iteration counter *i* exceeds predefined thresholds *N*, training is over.
**Algorithm 1** Pseudocode of the proposed method.
Input: $Train_{set},Test_{set}$, Initialized model parameter θOutput: Optimized model parameter $\theta^{\prime }$
 1: Loss *L* = 1, iteration number = *N*, counter *i* = 0, Loss threshold ϵ; 2: 
**while** (i>n) and (L<ϵ)
**do** 3:  
random select a batch of samples *S* from $Train_{set}$; 4:  
S=augment(S); 5:  
θ=Train(θ;S); 6:  
$L=Test(\theta ;Test_{set})$; 7:  
i++; 8: 
**end while**
 9: 
$\theta^{\prime }$ = θ; 10: 
**return**
$\theta^{\prime }$;

## 4. Experimental Evaluations

### 4.1. Experiment Setup

The generated benchmark dataset is used to evaluate the performance of heated metal mark attributes recognition, with deep convolutional neural networks models described in the above sections. In this case study, seven groups attributes of heated metal mark are considered. Each group of attributes are tested independently. Python is used as programming language. Tensorflow is adopted as deep learning framework and Keras is selected as the library. All the experiments are tested on Pentium i5-7 series CPU, 16G RAM, NvidiaGTX 1070 GPU, Ubuntu OS PC.

The experiments include the following aspects: (1) Evaluation of recognition rate with cross validation; (2) Evaluation of recognition efficiency; (3) Evaluation of different optimization method; (4) Evaluation of different batch size; (5) Evaluation of execution time.

### 4.2. Evaluation of Recognition Rate with Cross Validation

The recognition performance is evaluated independently for different attributes. Therefore, there are 7 groups of testing. The performance of heated metal mark image attribute recognition is computed with overall recognition rate, as shown in Equation (8). $N_{correct}$ denotes the number of correctly recognized samples. $N_{all}$ denotes the number of all testing samples. For each attribute, the dataset is divided into 5 subsets with attribute values equally distributed. 4 randomly chosen subsets (720 image samples) are used for training and the left subset (180 image samples) is used for testing. This process is repeated 4 times and the result is computed by averaging 4 independent testings.

(8) $$Recognition\;Rate=\frac{N_{correct}}{N_{all}}$$

Inception-v4, Inception-v3, ResNet, VGG16 and MobileNet are selected as basic CNNs architectures for evaluation. Factors of pre-trained model and data augment are considered. In this subsection, the pre-trained models are trained with COCO dataset [19]. If the pre-trained models are used for initialization, the parameters of low-level layers are fixed and the rest parameters are trainable. If the pre-trained models are not used for initialization, parameters of all layers are trainable. For data augment, commonly used transformations include random cropping, vertical and horizontal flipping, perturbation of brightness, saturation, hue and contrast are adopted. If the model is trained with data augment, 40% of training image in each batch are augmented, otherwise the probability is 10%. For model input, image size is set with 224×224×3 pixels. Epochs is set with 20 and batch size is set with 12. SGD is used as preferred optimization method. Learningrate is set with 0.0001 and momentum is set with 0.9. Dropout is set with 0.2.

The results of average recognition performance are shown in Table 3. Configurations of CNNs models, pre-trained model and data augment are listed in 1st, 2nd and 3rd columns respectively. The experiments are conducted under various condition combinations. $a_{i}$ means ith attribute. $train_{a}$ and $test_{a}$ denote recognition rate of training and testing. For convenience, $NetModel(p_{1},p_{2})$ is used to represent the model structure and parameters. NetModel∈{Inception-v4, Inception-v3, ResNet, VGG16, MobileNet}. $p_{1}$ and $p_{2}$ are parameters for pre-trained and data augment. $p_{i}\in \left\{0,1\right\}$, where 0 stands for off and 1 stands for on. For example, VGG16(0,1) means the model is trained with VGG16 structure, with pre-trained off and with data augment on.

For training performance, most models(with various configurations) finally reach 0.9 accuracy, and some are close to 1. Meanwhile, the training accuracy achieves stability after about 10 epochs for all attributes. The results demonstrate that the training accuracy for $a_{1}$ to $a_{7}$ are fine and acceptable. This is mainly because the CNNs models have relatively large scale, and with the significant ability of feature abstraction they get great recognition performance on training dataset. These results are similar with other research reports.

For testing performance, the experimental results show that Inception-v4(0,1) model gets top-1 performance on $a_{1}$ , with a value of 0.92. For $a_{2}$, Inception-v4(0,1) model gets top-1 result, with a value of 0.90. For $a_{3}$, Inception-v4(0,1) and ResNet(0,1) models get better results, with values of 0.83. For $a_{4}$, Inception-v3(0,1) gets top-1 result, with a value of 0.92. For $a_{5}$, Inception-v4(0,1) and Inception-v3(0,1) models get better results, with values of 0.92. For $a_{6}$, Inception-v4(0,1) and VGG16(0,1) models get better results, with values of 0.78. For $a_{7}$, Inception-v4(0,1), MobileNet(0,1) and Inception-v3(0,1) models get better results, with values of 0.91.

However, there are significant differences in testing accuracy versus epoch. Large fluctuations are shown especially on $a_{2}$, $a_{4}$ and $a_{7}$ in our experiments. This also reveal that different training modes have great influence on increasing the testing accuracy of models. Models with configuration (0,1) obtain better testing accuracy for all attributes. Unlike researches of other image recognition field that using pre-trained model can get better optimization, the experimental results in our study show divergences that heated metal mark image attribute recognition with pre-trained off gets best performances. The main reasons are that heated metal mark image is a very special research object, and there is large gap from the common image dataset, so the filters provided by pre-trained model obtained with common image dataset do not have much impact on our study. Inception-v4 outperforms other models on recognition performance demonstrates superiority of combining Inception and Residual. The result also shows that models with data augment can improve performance effectively. This is reasonable for training data with certain augment can increase the diversity of sample and model robust can be improved. It is especially important for large scale CNNs for its huge parameters are prone to overfitting with insufficient training data. However, among all tests only a small group of models achieve good convergence. The reason for this situation may originate from the complexity of this study, including uncertain noisy generated in process of training image generation or unsuitable attribute values definition. This can be solved by detailed model design and more careful tuning.

### 4.3. Evaluation of Recognition Efficiency

In this subsection, individual class recognition efficiency $\eta_{i}$, average recognition efficiency $\eta_{a}$ and the overall recognition efficiency $\eta_{o}$ are evaluated, which are defined in [44]. $q_{ij}$ is the number of samples of class *i* that was classified into class *j*. $n_{c}$ is the total number of classes and *N* is the total number of samples, as shown in Equations (9) and (10).

(9) $$\eta_{i}=\frac{q_{ii}}{\sum_{j=1}^{n_{c}}q_{ij}}$$

(10) $$\eta_{a}=\frac{\sum_{i=1}^{n_{c}}\eta_{i}}{n_{c}}$$

Table 4 gives the recognition efficiency of 5 different deep learning models. For convenient evaluating, all models are configured with (0,1). The results show that Inception-v4 gets optimal performance than other models which is coincide with results of Section 4.2. Moreover, individual efficiency $\eta_{i}$ has low variance and it also proves the stability of the model.

### 4.4. Evaluation of Optimization Method

In this subsection, performance of different optimization methods, Adam, Adagrad and SGD are evaluated. For comparison, Inception-v4(0,1) is used as basic CNNs model structure, and other parameters are the same as Section 4.2.

For the training process, Adam and SGD get better results on attributes $a_{1}$, $a_{2}$, $a_{3}$, $a_{5}$, $a_{7}$. SGD gets best result on attributes $a_{4}$ and $a_{6}$. While, Adagrad gets worst result on all attributes. For the testing process, the results are the same as the training process. This phenomenon may be originated from the reason that SGD is simple but always works well for most tasks, and Adam and Adagrad are more fragile in our study and it may needs more complex tuning.

### 4.5. Evaluation of Batch Size

Batch size is another important factor for training CNNs model. In this subsection, performance of different batch sizes, 8, 12, 16, 24 and 32 are evaluated. For comparison, Inception-v4(0,1) is used as basic CNNs model structure, and other parameters are the same as Section 4.2.

For training process, models with batch size = 8 convergences slow, especially on attributes $a_{1}$, $a_{2}$ and $a_{4}$. Models with batch size = 12, 16, 24, 32 get good results. For testing process, we can see that large batch size leads to better model training and generalization. Comparing with models trained in Section 4.2 (batch size = 12), the performances of models trained with batch size = 32 are improved with 0.5%, 0.8%, 0.5%, 0.7%, 0.8%, 2.5%, 0.9% for attributes $a_{1}$ to $a_{7}$ respectively. There is obvious performance enhancement for attribute $a_{6}$, while there are not much improvements for other attributes.

### 4.6. Evaluation of Execution Times

In this subsection, model execution time is evaluated. Training and testing time of 5 deep leaning models with various batch sizes (8, 12, 16, 24 and 32) are tested. Table 5 shows that VGG16 model cost the most execution time, with 2.67s, 3.05 s, 3.33 s, 3.67 s and 4.33 s for batch size of 8, 12, 16, 24 and 32 for each training iteration. MobiliNets gets the least execution time, with about 60% of VGG16’s. Inception-v4 is the preferred model for its excellent performance and acceptable cost. For testing time, MobiliNets has the minimal cost, 0.031s. Moreover, trade off is feasible according to diverse needs.

## 5. Conclusion and Future Works

Heated metals are usually retained at the fire scene, and their mark can be used as an important trace for fire analysis. Traditional methods recognize attribute of heat metal mark mainly depend on human expert with knowledge of physics and chemistry. This makes the work very difficult to popularize and automate. This paper makes a case study on heated metal mark image attribute recognition based on convolutional neural networks. The benchmark dataset for training and testing is designed. For seven selected attributes, various CNNs architectures, parameters, training mode, recognition efficiency and execution time are evaluated and analyzed. One of the greatest advantages of this work is that the feasibility of attribute recognition on heated metal mark image based on top CNNs models is studied. Through the study, we can conclude that it is possible to recognise attributes of heated metal mark using the tuned deep CNNs model with an acceptable accuracy, and it can be implemented for real time applications.

This study needs to be further improved. Our future works will focus on three aspects: (1) More datasets will be generated to enrich the benchmarks; (2) recognition with multi-atrributes joint model will be studied and (3) Works will be focus on customed CNNs models structure design, and more fine-tuning techniques will be developed.

## Figures and Tables

**Table 1:** Relations between color change of ferrous metal and heating temperature. The heating duration time is set with 30 min.

Color	Heating Temperature
dark purple	300 ${}^{\circ }$C
sky blue	350 ${}^{\circ }$C
brown	450 ${}^{\circ }$C
dark red	500 ${}^{\circ }$C
orange	650 ${}^{\circ }$C
light yellow	1000 ${}^{\circ }$C
white	1200 ${}^{\circ }$C

**Table 2:** Attributes of heated metal defined in this study.

Attribute Abbr.	Attribute Name	Types (Predefined Label Values)
$a_{1}$	Metal type	2 types: (1) galvanized steel; (2) cold rolled steel
$a_{2}$	Heating mode	3 types: (1) vacuum; (2) muffle furnace; (3) gasoline burner
$a_{3}$	Heating temperature	4 degrees: (1) 400 ${}^{\circ }$C; (2) 600 ${}^{\circ }$C; (3) 800 ${}^{\circ }$C; (4) 1000 ${}^{\circ }$C
$a_{4}$	Heating duration	4 degrees: (1) 15 min; (2) 30 min; (3) 40 min; (4) 45 min
$a_{5}$	Cooling mode	2 types: (1) Natural cooling; (2) forced cooling
$a_{6}$	Placing duration	3 degrees: (1) 24 h; (2) 36 h; (3) 48 h
$a_{7}$	Relative humidity	2 degrees: (1) 65%; (2) 85%

**Table 3:** Recognition rate of heated metal mark attributes.

Model	Pre-Trained	Data Augment	$a_{1}$		$a_{2}$		$a_{3}$		$a_{4}$		$a_{5}$		$a_{6}$		$a_{7}$	
			${\mathrm{train}}_{a}$	${\mathrm{test}}_{a}$	${\mathrm{train}}_{a}$	${\mathrm{test}}_{a}$	${\mathrm{train}}_{a}$	${\mathrm{test}}_{a}$	${\mathrm{train}}_{a}$	${\mathrm{test}}_{a}$	${\mathrm{train}}_{a}$	${\mathrm{test}}_{a}$	${\mathrm{train}}_{a}$	${\mathrm{test}}_{a}$	${\mathrm{train}}_{a}$	${\mathrm{test}}_{a}$
Inception-$v_{4}$	yes	no	0.96	0.58	0.98	0.27	0.97	0.81	0.98	0.20	0.98	0.33	0.98	0.41	0.98	0.31
	yes	yes	0.98	0.45	0.99	0.30	0.94	0.69	0.98	0.48	0.98	0.82	0.98	0.40	0.98	0.57
	no	no	0.98	0.72	0.92	0.82	0.99	0.80	0.95	0.49	0.99	0.85	0.98	0.62	0.91	0.83
	no	yes	0.96	0.92	0.98	0.90	0.98	0.83	0.97	0.85	0.99	0.92	0.97	0.78	0.95	0.91
Inception-$v_{3}$	yes	no	0.96	0.60	0.98	0.25	0.99	0.81	0.97	0.47	0.99	0.80	0.96	0.47	0.92	0.31
	yes	yes	0.99	0.72	0.97	0.40	0.99	0.81	0.99	0.53	0.99	0.87	0.99	0.54	0.99	0.34
	no	no	0.97	0.79	0.90	0.63	0.94	0.82	0.93	0.60	0.99	0.86	0.85	0.59	0.95	0.48
	no	yes	0.97	0.90	0.96	0.85	0.99	0.81	0.98	0.91	0.99	0.92	0.97	0.69	0.98	0.91
ResNet	yes	no	0.98	0.56	0.98	0.53	0.99	0.80	0.99	0.15	0.98	0.67	0.98	0.40	0.98	0.24
	yes	yes	0.98	0.45	0.98	0.43	0.99	0.79	0.97	0.26	0.99	0.67	0.98	0.39	0.98	0.39
	no	no	0.90	0.72	0.94	0.41	0.92	0.80	0.94	0.48	0.99	0.68	0.85	0.61	0.93	0.73
	no	yes	0.93	0.89	0.90	0.83	0.94	0.83	0.94	0.65	0.98	0.73	0.94	0.73	0.91	0.90
VGG16	yes	no	0.92	0.72	0.95	0.71	0.90	0.20	0.92	0.64	0.99	0.69	0.92	0.77	0.90	0.52
	yes	yes	0.96	0.68	0.97	0.65	0.98	0.21	0.96	0.49	0.99	0.74	0.96	0.60	0.94	0.55
	no	no	0.93	0.61	0.92	0.63	0.90	0.32	0.91	0.51	0.98	0.74	0.66	0.63	0.95	0.81
	no	yes	0.93	0.87	0.90	0.85	0.94	0.81	0.93	0.72	0.98	0.80	0.83	0.78	0.97	0.89
MobileNets	yes	no	0.98	0.58	0.97	0.43	0.98	0.21	0.98	0.57	0.98	0.81	0.98	0.49	0.98	0.13
	yes	yes	0.97	0.73	0.98	0.55	0.99	0.38	0.98	0.66	0.98	0.79	0.97	0.61	0.99	0.34
	no	no	0.90	0.68	0.91	0.75	0.94	0.82	0.98	0.53	0.99	0.81	0.85	0.65	0.95	0.55
	no	yes	0.93	0.90	0.92	0.83	0.96	0.82	0.90	0.82	0.99	0.89	0.91	0.74	0.97	0.91

**Table 4:** Classification efficiency analysis.

Attribute	Efficiency	Inception-v4	Inception-v3	ResNet	VGG16	MobileNets
$a_{1}$	$\eta_{1}$	0.93	0.87	0.88	0.89	0.91
	$\eta_{2}$	0.91	0.93	0.90	0.85	0.89
	$\eta_{a}$	0.92	0.90	0.89	0.87	0.90
$a_{2}$	$\eta_{1}$	0.92	0.83	0.79	0.82	0.80
	$\eta_{2}$	0.91	0.84	0.84	0.86	0.84
	$\eta_{3}$	0.87	0.88	0.83	0.87	0.85
	$\eta_{a}$	0.90	0.85	0.82	0.85	0.83
$a_{3}$	$\eta_{1}$	0.80	0.78	0.79	0.77	0.80
	$\eta_{2}$	0.83	0.85	0.86	0.84	0.83
	$\eta_{3}$	0.85	0.80	0.83	0.83	0.80
	$\eta_{4}$	0.84	0.81	0.84	0.81	0.85
	$\eta_{a}$	0.83	0.81	0.83	0.81	0.82
$a_{4}$	$\eta_{1}$	0.80	0.88	0.60	0.68	0.80
	$\eta_{2}$	0.83	0.92	0.68	0.76	0.85
	$\eta_{3}$	0.87	0.90	0.70	0.75	0.79
	$\eta_{4}$	0.86	0.93	0.63	0.69	0.84
	$\eta_{a}$	0.85	0.91	0.65	0.72	0.82
$a_{5}$	$\eta_{1}$	0.90	0.89	0.71	0.83	0.88
	$\eta_{2}$	0.94	0.95	0.75	0.77	0.90
	$\eta_{a}$	0.92	0.92	0.73	0.80	0.89
$a_{6}$	$\eta_{1}$	0.75	0.66	0.70	0.73	0.71
	$\eta_{2}$	0.77	0.73	0.72	0.80	0.75
	$\eta_{3}$	0.82	0.68	0.77	0.81	0.76
	$\eta_{a}$	0.78	0.69	0.73	0.78	0.74
$a_{7}$	$\eta_{1}$	0.87	0.90	0.88	0.87	0.88
	$\eta_{2}$	0.95	0.92	0.92	0.91	0.94
	$\eta_{a}$	0.91	0.91	0.90	0.89	0.91

**Table 5:** Execution time (seconds).

Execution Time	Batch Size	VGG16	Inception-v4	Inception-v4	ResNet	MobileNets
Training time	8	2.67	2.45	2.03	1.84	1.59
	12	3.05	2.77	2.29	2.09	1.81
	16	3.33	3.08	2.54	2.31	2.04
	24	3.67	3.41	2.81	2.54	2.23
	32	4.33	4.05	3.34	3.02	2.65
Testing time	x	0.11	0.083	0.062	0.045	0.031
